# Exploring Enzymatic Hydrolysis of Urine Samples for Investigation of Drugs Associated with Drug-Facilitated Sexual Assault

**DOI:** 10.3390/ph17010013

**Published:** 2023-12-21

**Authors:** Kathrine Skov, Sys Stybe Johansen, Kristian Linnet, Brian Schou Rasmussen, Marie Katrine Klose Nielsen

**Affiliations:** Section of Forensic Chemistry, Department of Forensic Medicine, University of Copenhagen, 2200 Copenhagen, Denmark; sys.johansen@sund.ku.dk (S.S.J.); kristian.linnet@sund.ku.dk (K.L.); brian.rasmussen@sund.ku.dk (B.S.R.); marie.nielsen@sund.ku.dk (M.K.K.N.)

**Keywords:** glucuronides, DFSA, deconjugation, β-glucuronidase, drug stability, recombinant enzyme

## Abstract

Analyzing urine is common in drug-facilitated sexual assault cases if the analysis of blood is not optimal. The efficient enzymatic pretreatment of urine is important for cleaving glucuronides and improving the detection of the parent drug. The aim was to investigate the efficiency of three β-glucuronidases on eleven glucuronides relevant to DFSA at different incubation periods and temperatures. Human drug-free urine was fortified with 11 glucuronides, hydrolyzed with either β-glucuronidase/arylsulfatase (*Helix Pomatia*), recombinant β-glucuronidase B-One™ or recombinant β-glucuronidase BGTurbo™ and incubated for 5, 10, 60 min, 18 h and 24 h at 20 °C/40 °C/55 °C before UHPLC–MS/MS analysis. The stability of 141 drugs and metabolites relevant to DFSA was investigated by incubating fortified urine under the same hydrolysis conditions. B-One™ showed efficient hydrolysis (>90%) of most glucuronides in 5 min at all temperatures, while BGTurbo™ showed a similar efficiency (>90%), but the optimal temperature (20–55 °C) and incubation time (5–60 min) varied among analytes. The β-glucuronidase/arylsulfatase had the lowest efficiency and required the longest incubation (24 h) at 40–55 °C. The stability of 99% of 141 drugs and metabolites was not affected by incubation at 20–55 °C for 24 h. Recombinant enzymes show promising results for the simple and efficient hydrolysis of a broad panel of glucuronides relevant for DFSA.

## 1. Introduction

Drug-facilitated sexual assaults (DFSA) are sexual acts towards an individual who is incapacitated due to the influence of intoxicating substances [1,2]. Unfortunately, DFSA cases are often unreported or reported too late to detect any drugs in blood or urine. With increasing time delay from assault to medical examination, the drugs are more likely to have been eliminated from the body. Consequently, rapid sample collection is vital to ensure forensic evidence that the victim was under the influence of intoxicating substances during the assault. The amount of samples with positive toxicology decreases considerably when the time delay from assault to sample collection exceeds 12–24 h [3,4,5,6,7]. Furthermore, victims of DFSA report the assault later compared to victims of other sexual assaults [8], which is likely a result of the sedative, hypnotic, and/or anterograde amnesic effects caused by the drugs typically implicated in DFSA. Additionally, victims might hesitate to report the assault for several other reasons, such as embarrassment, guilt, or discouragement [1,2].

A multitude of substances have the potential to be used to facilitate sexual assault, but ethanol, drugs of abuse, and benzodiazepines are most frequently observed in DFSA cases [1,2,6,9,10,11]. However, some drugs, such as gamma-hydroxybutyrate (GHB) and Z-drugs (zolpidem, zopiclone, and zaleplon), are particularly challenging to detect due to their short half-life [12]. The rate of drug metabolism and elimination determines the chances of drug detection, and the analysis of urine samples generally allows for a longer detection window compared to blood owing to the presence of metabolites. Metabolism facilitates the excretion of drugs through two metabolic pathways. In Phase-I-metabolism, the drug is modified through reduction, oxidation, and hydrolysis reactions, while in Phase-II-metabolism, conjugation reactions increase hydrophilicity and allow for renal excretion [13,14]. The resulting conjugated metabolites usually have a longer half-life than the parent drug and thus pre-analytical deconjugation is typically implemented to enhance drug detection. This hydrolysis process cleaves glucuronide and/or sulfate conjugated metabolites and enables the detection of the parent drug [15,16]. Various sources of β-glucuronidases (mollusks, bovine, *Escherichia coli*) are used but, recently, recombinant enzymes have shown promising results regarding efficient and fast hydrolysis at lower incubation temperatures compared to β-glucuronidases from other sources [17,18]. A previous study reported on the efficient use of a recombinant enzyme on the hydrolysis of three benzodiazepine glucuronides [17]. Additionally, another study reported that enzyme efficiency varied across pH and that the optimum pH for hydrolysis varied according to the individual glucuronide substrates. While several β-glucuronidases exerted hydrolysis activity towards a range of common glucuronides, the individual activity profile was subject to substrate bias [19]. Thus, enzyme efficiency varies with regard to analytes [18,19] and their effective performance across a broader panel of glucuronides relevant to DFSA needs further investigation.

Sensitive analytical methods are a fundamental part of forensic toxicology and the effective hydrolysis of conjugated metabolites such as glucuronides is important for the improved detection of drugs in urine analysis [19]. Consequently, the continuous advancement of analytical methods is essential to ensure that fewer drugs go undetected in DFSA cases. The objective of this study was to investigate the optimal hydrolysis for the pretreatment of urine samples to optimize the systematic toxicological analysis of urine samples from DFSA cases. To achieve this, we investigated the efficiency of three commercially available enzymes on the hydrolysis of a broad panel of 11 conjugated metabolites typically associated with DFSA. Furthermore, we examined the stability of 141 selected drugs and metabolites under different hydrolysis incubation temperatures and time periods.

## 2. Results and Discussion

### 2.1. Hydrolysis Efficiency

**Figure 1:**
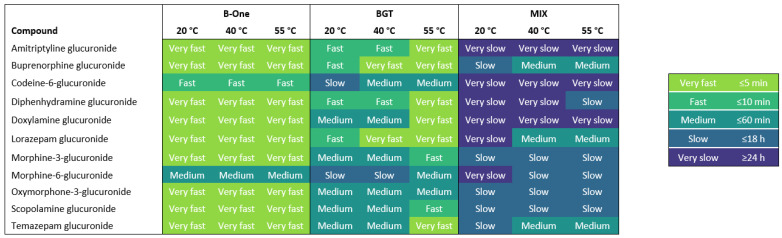
Heatmap showing the hydrolysis efficiency of the enzymes B-One, BGT and MIX on the 11 glucuronides. The hydrolysis efficiency is based on ≥90% cleavage and ranked by color, with the fastest efficiencies being lighter in color and the slowest efficiencies being darker in color.

The hydrolysis efficiency was evaluated for three β-glucuronidases on 11 different glucuronide metabolites. These glucuronides were chosen based on their relevance to DFSA and their commercial availability. The hydrolysis efficiency was found by assessing how fast more than 90% cleavage was obtained for each of the 11 glucuronides under each enzymatic treatment setup (Figure 1).

Generally, B-One showed the best overall hydrolysis efficiency of most glucuronides, reaching ≥90% cleavage within 5 min (Figure 1). The hydrolysis of morphine-6-glucuronide and codeine-6-glucuronide was slightly longer than the rest of the analytes, occurring in 10 min and 60 min, respectively. BGT showed a similar efficiency to B-One, but glucuronide cleavage was faster at 55 °C for most analytes (Figure 1). The hydrolysis was least efficient for codeine-6-glucuronide and morphine-6-glucuronide with 90% cleavage being reached within 18 h at 20 °C. However, the efficiency improved to ≥90% cleavage within ≤60 min when increasing incubation temperature to 40 °C and 55 °C. Thus, BGT showed fast (5–60 min) and efficient hydrolysis, but the optimal temperature and incubation time was more analyte-specific, which was also noted in the manufacturer instructions. The MIX enzyme showed the lowest overall hydrolysis efficiency (Figure 1). With this enzymatic treatment, an incubation time of either 18 h or more than 24 h was needed to reach 90% cleavage for most glucuronides. The hydrolysis was least efficient for amitriptyline glucuronide, codeine-6-glucuronide and doxylamine glucuronide at all temperatures. Furthermore, the enzyme was most efficient at hydrolyzing buprenorphine glucuronide, lorazepam glucuronide and temazepam glucuronide at 40 °C and 55 °C. It should be noted that the estimated efficiency does not take possible enzyme breakdown into consideration. Thus, it is plausible that incubation for 24 h at 55 °C could affect an enzyme like MIX, which has optimal conditions at 37–40 °C.

**Figure 2:**
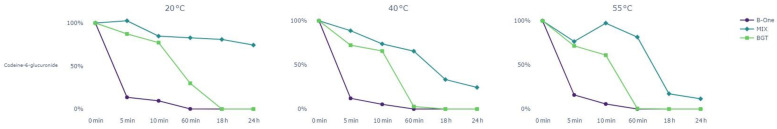
Cleavage (%) of codeine-6-glucuronide when enzymatically hydrolyzed with either MIX, B-One or BGT at 20 °C, 40 °C and 55 °C for 24 h. The *x*-axis is in categorical order based on the five incubation periods.

**Figure 3:**
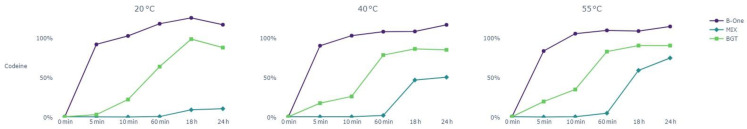
Analyte recovery (%) for codeine when its corresponding glucuronide is enzymatically hydrolyzed with either MIX, B-One or BGT at 20 °C, 40 °C and 55 °C for 24 h. The *x*-axis is in categorical order based on the five incubation periods.

An illustrative example of glucuronide cleavage and parent drug formation, expressed as recovery for all incubation periods, temperatures, and enzymes, is shown in Figure 2 and Figure 3. The cleavage of all 11 glucuronides and the recovery of their 10 parents at all incubation periods and temperatures is illustrated in Supplementary Figures S1 and S2. B-One cleaved 91% of codeine-6-glucuronide within 10 min and a maximum hydrolysis of >99% was reached within 60 min independent of temperature (Figure 2). A corresponding codeine recovery was observed at 10–60 min (Figure 3). For BGT, maximum hydrolysis (>99%) and recovery (91%) were reached after 60 min at 55 °C. In comparison, the treatment with MIX resulted in maximum hydrolysis of codeine-6-glucuronide (88%) and maximum codeine recovery (75%) after 24 h at 55 °C. The discrepancy between maximum hydrolysis and maximum recovery of up to 20% may be due to differences in the purity of the reference standard, the choice of internal standard, the limits of quantification, and analytical uncertainty.

Generally, B-One displayed the best hydrolysis efficiency by cleaving most glucuronide linkages (>90%) in 5 min independent of temperature, while the efficiency of BGT and MIX were more analyte-specific and increased with increasing temperature and incubation periods (Supplementary Tables S3–S5). Hydrolysis efficiency is expected to vary among analytes due to their varied chemical structure. As such, it has previously been shown that β-glucuronidases exert a preferential hydrolysis of *O*-glucuronides over *N*-glucuronides. Additionally, *O*-glucuronides can be more labile under basic conditions, while *N*-glucuronides can be more labile under acidic conditions [18,20,21]. In the present study, the recombinant enzymes B-One and BGT efficiently hydrolyzed the *N*-glucuronides amitriptyline glucuronide (>99%), diphenhydramine glucuronide (>99%) and doxylamine glucuronide (>99%) within 5–10 min (Figure 1). While the efficiency of B-One was independent of temperature, the efficiency of BGT improved at 40 °C and 55 °C. In contrast, the MIX enzyme was less efficient in hydrolyzing these three glucuronides and generally needed to be incubated for a longer time compared to B-One and BGT (Figure 1).

It has previously been shown that β-glucuronidase from Helix pomatia was more efficient in hydrolyzing morphine-3-glucuronide than morphine-6-glucuronide and codeine-6-glucuronide [22]. In this study, MIX exerted the complete hydrolysis of morphine-3-glucuronide (100%) and morphine-6-glucuronide (98%), but less so with codeine-6-glucuronide (76–88%) (Supplementary Tables S3–S5). Correspondingly, a longer incubation time was needed for codeine-6-glucuronide (Figure 1). Both recombinant β-glucuronidases showed the efficient hydrolysis of morphine-3-glucuronide (100%), morphine-6-glucuronide (>99%) and codeine-6-glucuronide (>99%). However, the hydrolysis of morphine-3-glucuronide was faster (5–10 min) than the hydrolysis of morphine-6-glucuronide (10–60 min) and codeine-6-glucuronide (10–60 min) for both recombinant enzymes (Figure 1).

Analyte recovery was good (70–100%) for most of the parent drugs (Supplementary Tables S3–S5). However, the recovery was low for doxylamine (39–40%) and oxymorphone (16–24%), which might be due to lower purity of the glucuronide reference standard, which was less than 100%. This was independent of the enzyme used but, ultimately, the recombinant enzymes still presented the highest recovery.

In the present study, we demonstrate that recombinant enzymes enable the efficient hydrolysis of a broader panel of glucuronides relevant to DFSA. Similarly, Morris et al. compared a recombinant enzyme with a β-glucuronidase from Abalone and found that the recombinant enzyme efficiently hydrolyzed lorazepam glucuronide, oxazepam glucuronide, and temazepam glucuronide at room temperature [17]. A comparison of recombinant and non-recombinant enzymes has also been carried out concerning the hydrolysis of urinary conjugates of triclocarban, parabens, and phenols [18]. Lee et al. reported that β-glucuronidases exert different pH and substrate profiles, indicating that enzyme efficiency varies across glucuronides [19]. Similarly, we observed that the efficiency of the tested β-glucuronidases was analyte-specific. However, the recombinant enzyme B-One showed the best overall efficiency across the entire panel of glucuronides, indicating its usefulness in the pretreatment of urine samples analyzed for a broad range of compounds. The screening of a multitude of compounds is especially relevant in cases of DFSA, whereby many drugs, pharmaceuticals and over-the-counter medications can be misused to facilitate sexual assault.

### 2.2. Stability of Drugs during Incubation

**Figure 4:**
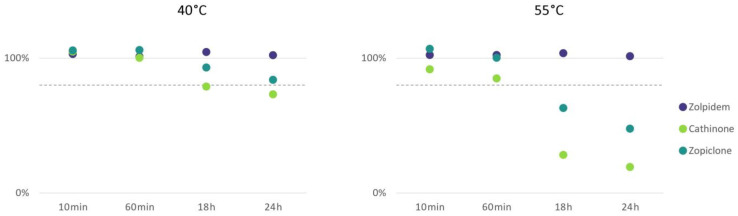
Average decrease (%) of cathinone and zopiclone peak area during incubation at 40 °C and 55 °C for 24 h. Zolpidem is shown as an example of 1 of the 139 DFSA-relevant drugs where stability was not affected. The dotted line represents the −20% limit. Each experiment was performed in duplicate.

The stability of 141 drugs and metabolites relevant to DFSA was examined during incubation for 24 h at 20 °C, 40 °C and 55 °C to evaluate whether the typical pretreatment of urine samples causes considerable analyte loss. The results showed that the incubation of 99% drugs relevant to DFSA did not show a decline of more than 20%. However, cathinone and zopiclone showed considerable declines (52% and 81%) during incubation for 24 h at 40 °C and 55 °C (Figure 4). Cathinones in urine have been shown to be pH- and temperature-dependent with elevated temperatures causing more alkaline urine and thus more instability [23,24,25]. Additionally, the instability of zopiclone is well-documented in blood and in urine, as well as its degradation into ACP [26,27,28,29,30]. The pH of urine typically increases with increasing storage temperature due to microbial growth [23,24,25], but preservation with sodium fluoride, storage in a freezer and the addition of a buffer during pretreatment should typically keep the pH of the urine samples stable. However, it is plausible that the stability of zopiclone and cathinone was affected by other factors such as light or oxidation [31].

Lorazepam showed a decline of less than 20%, but a decrease was observed after 60 min at 55 °C during all three enzymatic treatments of lorazepam glucuronide (Supplementary Figure S2). This indicates that lorazepam might experience some instability, though still less than a 20% decline. Thus, using an enzyme that requires short incubation time at room temperature will both decrease the sample preparation time and likely decrease analyte loss during enzymatic pretreatment. Providing an efficient hydrolysis while ensuring a gentle sample treatment is especially important in DFSA cases where high sensitivity is needed to ensure the detection of single-dose concentrations hours or days after exposure.

### 2.3. Considerations for Choice of Enzyme

The drug and metabolite concentration will likely be low in many cases of DFSA if the assault is reported late. Thus, improving enzymatic hydrolysis can potentially increase sensitivity in the detection of any drugs present in urine. In this study, we showed that efficient and rapid hydrolysis can be obtained by using recombinant β-glucuronidases for the hydrolysis of a broad range of glucuronides. However, while B-One and BGT only had β-glucuronidase activity, the MIX enzyme had both β-glucuronidase and arylsulfatase activity. This could potentially make the MIX enzyme more useable on a broader panel of drugs. As such, it is important to consider the case and which analytes to investigate before choosing the appropriate enzyme for the hydrolysis of urine samples. Thus, to extensively cover most drugs relevant to DFSA, it could also be appropriate to screen directly for some metabolites, such as sulfate conjugates, which would require sensitive analytical methods. Among the drugs relevant to DFSA, duloxetine, and paroxetine are metabolized into both glucuronide and sulfate conjugates. For paroxetine, the sulfate metabolite is excreted to a higher degree (17%) than the glucuronide metabolites (3.1–8%) [32]. Similarly, duloxetine is excreted as its methyl, glucuronide and sulfate metabolites [33]. Consequently, it would be important to consider both of their sulfate metabolites in the methodology when analyzing samples from DFSA cases.

It is important to take several things into consideration when establishing an appropriate workflow for the analysis of urine samples. The economy, equipment, workload, and sample throughput determine the choice of method. Recombinant enzymes provide rapid and more efficient results but can be expensive in laboratories with a high throughput of samples. While the enzyme from *H. pomatia* required longer incubation time and heating, the product costs per sample were considerably lower. As such, the choice of enzyme might also be dependent on a cost–benefit analysis for the individual laboratory. Recombinant enzymes provide a rapid hydrolysis that decreases the risk of analyte loss during incubation. However, if using an enzyme that requires heated incubation for longer periods, including an unhydrolyzed urine sample in addition to a hydrolyzed sample to evaluate any potential analyte loss is also a possibility. Another important thing to note is that the efficiency of some enzymes seems to be analyte-dependent. In this study, B-One showed a similar efficiency on a broad panel of metabolites, while the efficiency of BGT and MIX was more analyte-dependent. Therefore, choosing the optimal enzyme depends entirely on the requirements of the given laboratory and the objective of the analysis for which the samples are prepared.

## 3. Materials and Methods

### 3.1. Chemicals and Reagents

The following reference standards were purchased from Toronto Research Chemicals (Toronto, Canada): amitriptyline-N-β-D-glucuronide (97%), codeine-6-glucuronide (96%), diphenhydramine-N-β-D-glucuronide (94%), doxylamine-β-D-glucuronide (96%), lorazepam (98%), scopolamine-β-glucuronide (>85%), scopolamine (98%) and oxymorphone-3-glucuronide (96%). Diphenhydramine (99.9%) and morphine-3-glucuronide (92.8%) were obtained from Sigma-Aldrich (St. Louis, MI, USA). Morphine (98.5%), morphine-6-glucuronide (98%) and temazepam glucuronide (98.5%) were acquired from Lipomed GmbH (Arlesheim, Switzerland). Amitriptyline (99%), buprenorphine (99.9%), buprenorphine glucuronide (99%), codeine (99%), oxymorphone (>99%) and temazepam (99%) were purchased from Cerilliant (Round Rock, TX, USA). Lorazepam glucuronide (99.7%) was obtained from Merck (Darmstadt, Germany) and doxylamine (100%) was obtained from U.S. Pharmacopeia (Rockville, MD, USA).

The internal standards amitriptyline-d_6_, codeine-d_6_, morphine-d_6_, oxazepam-d_5_ and temazepam-d5 were obtained from CDN Isotopes (Pointe-Claire, Canada), Toronto Research Chemicals (Toronto, Canada), Cerilliant (Round Rock, TX, USA) and Lipomed GmbH (Arlesheim, Switzerland).

LC–MS grade acetonitrile (≥99.9%), methanol (≥99.9%) and deionized water were purchased from Fisher Scientific (Loughborough, Leicestershire, UK). Formic acid (98–100%) was obtained from Merck (Darmstadt, Germany). The reconstitution solvent comprised methanol, acetonitrile and 0.1% formic acid in water (6.25/6.25/87.5, *v*/*v*/*v*).

### 3.2. Enzymes

A β-glucuronidase/arylsulfatase (annotated as MIX) from Helix Pomatia was purchased from Roche (Mannheim, Germany) with β-glucuronidase activity ≥100,000 U/mL and sulfatase activity 800,000 Roy units. Recombinant β-glucuronidase B-One™ (annotated as B-One) with β-glucuronidase activity ≥12,000 Product Specific-Units/mL and BGTurbo™ (annotated as BGT) recombinant β-glucuronidase with β-glucuronidase activity ≥200,000 U/mg protein were obtained from Kura Biotech Inc (Puerta Varas, Chile). The buffer for BGT was prepared by dissolving the accompanying instant buffer into deionized water. The buffer for the MIX enzyme was prepared as 4% ammonium acetate and 3% acetic acid in water. The recombinant enzyme B-One was purchased as a combined enzyme and buffer solution.

### 3.3. Preparation of Standard Solutions

Glucuronides were prepared individually as stock standard solutions (1 mg/L) in either methanol or dimethyl sulfoxide. A stock solution of 11 glucuronides was prepared in 50% methanol in water at concentrations ranging from 2.5 to 20.0 mg/L, depending on the drug, and stored at −20 °C. A stock solution containing parent drugs to the 11 glucuronides was prepared in 50% methanol in water at a concentration of 5 mg/L and stored at −20 °C. For stability testing, an additional stock solution containing 141 drugs and metabolites relevant to DFSA was prepared in 50% methanol in water at concentrations ranging from 5.0 to 250 mg/L. Six standard spiking solutions were prepared from serial dilutions of the glucuronide stock solution. An additional six working standard spiking solutions were prepared by dilutions of the parent drug stock solution and the stock solution containing 141 DFSA drugs. An internal standard (IS) solution of deuterated ISs was prepared in 50% methanol in water at concentrations of 0.016–0.05 mg/L and stored at −80 °C.

A neat standard solution containing 11 glucuronides at concentrations of 0.05–1.8 mg/L, and a neat standard solution containing their parent drugs at concentrations of 0.04–8.00 mg/L and ISs, were prepared in reconstitution solvent and used to verify retention times (RT) in LC–MS/MS.

### 3.4. Fortified Urine Pools and Calibrators

Pooled human drug-free urine from volunteers was stored at −20 °C in vacutainers with sodium fluoride and potassium oxalate. For each enzyme, three urine pools (3 × 1500 µL) comprising drug-free urine and manufacturer-recommended buffer were prepared and fortified with glucuronide stock solution and IS. The concentrations in urine were 0.14 mg/L for amitriptyline glucuronide, 0.55 mg/L for buprenorphine glucuronide, 0.22 mg/L for codeine-6-glucuronide, 0.55 mg/L for diphenhydramine glucuronide, 0.55 mg/L for doxylamine glucuronide, 0.13 mg/L for lorazepam glucuronide, 1.11 mg/L for morphine-3-glucuronide, 1.11 mg/L for morphine-6-glucuronide, 1.11 mg/L for oxymorphine-3-glucuronide, 1.11 mg/L for scopolamine glucuronide and 0.14 mg/L for temazepam glucuronide. The fortified urine pools were prepared with urine:buffer ratios (MIX: 1:10; B-One: 2:1; BGT: 2:5) according to the manufacturer instruction sheets.

To assess the stability of 141 drugs and metabolites, an additional three fortified urine pools (3 × 1500 µL) comprising drug-free urine, internal standards, buffer, and stock solution of 141 drugs and metabolites was prepared. The concentration of the 141 drugs and metabolites in urine ranged from 0.04 to 8.8 mg/L.

Six standard spiking solutions were used to prepare a calibration curve in human drug-free urine with glucuronide concentrations ranging from 0.0025 to 9.0 mg/L depending on individual drug concentration. Another six standard spiking solutions with parent drugs were used to prepare a calibration curve in human drug-free urine with concentrations ranging from 0.14 to 5.0 mg/L depending on the concentration of each drug.

### 3.5. Hydrolysis of Urine Samples

Enzyme (MIX: 70 µL; B-One: 900 µL; BGT: 200 µL) was added to the three fortified urine pools, and they were incubated at either 20 °C, 40 °C or 55 °C, respectively. Duplicate aliquots (140 µL) were transferred from the fortified urine pools to individual sample tubes after 5 min, 10 min, 60 min, 18 h and 24 h, respectively. This setup was repeated for each enzyme. Immediately following the designated incubation period, enzymatic hydrolysis was stopped with the addition of acetonitrile (140 µL). The samples were centrifuged (3600 rpm for 10 min) and 200 µL supernatant was collected for evaporation to dryness using nitrogen at 40 °C. The residue was reconstituted in 100 µL reconstitution solvent and the supernatant was transferred to an HPLC vial with an insert for analysis. A duplicate of glucuronide-fortified urine at time 0 min was included to compare with the theoretical spiked concentration. Furthermore, triplicates of blank urine incubated at 20 °C, 40 °C or 55 °C for 24 h were included as interference controls.

### 3.6. Testing Stability during Incubation

Three urine pools fortified with 141 drugs and metabolites were incubated at 20 °C, 40 °C or 55 °C, respectively. Duplicate aliquots (140 µL) were transferred from the fortified urine pools to individual sample tubes after 5 min, 10 min, 60 min, 18 h and 24 h. Acetonitrile was added to each individual sample (140 µL) and samples were centrifuged (3600 rpm for 10 min). The supernatant (200 µL) was collected for evaporation to dryness using nitrogen at 40 °C and the residue was reconstituted in 100 µL reconstitution solvent prior to being transferred to an HPLC vial with an insert for analysis.

### 3.7. Quantification by UHPLC–MS/MS

**Figure 5:**
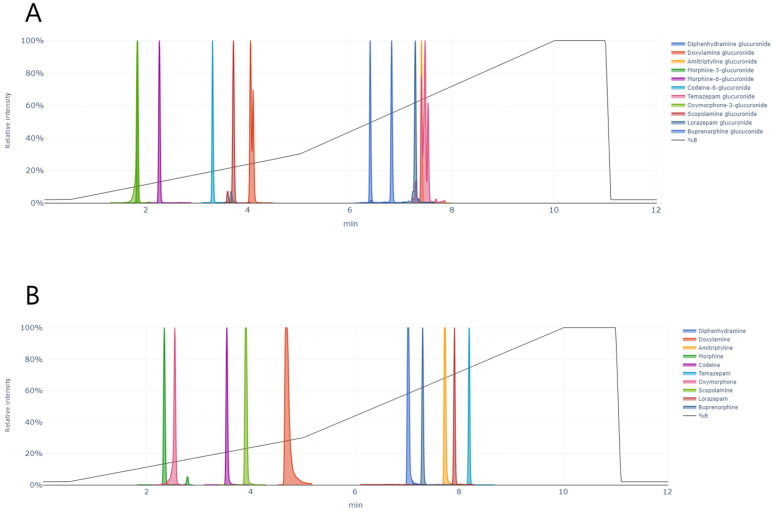
Ion chromatograms of (**A**) 11 glucuronides and (**B**) parent drugs of the glucuronides in an extracted urine sample and the solvent gradient (2–100% B).

Sample analysis was performed on a Waters ACQUITY UPLC system coupled to an Xevo TQS tandem mass spectrometer (Waters, Milford, MA, USA). Analyte separation was performed on an ACQUITY UPLC HSS T3 column (100 × 2.1 mm, 1.8 µm) at 45 °C. The mobile phase consisted of A) 1 mM ammonium formate in 0.1% FA and B) 0.1% FA in methanol and acetonitrile (1:1). The flow rate was 0.40 mL/min, and the gradient was 2% B (0–5 min), 30% B (5–10 min), 100% B (10–11.1 min) and 2% B (11.1–12 min). The autosampler temperature was 10 °C to ensure analyte stability and the injection volume was 0.4 µL. All glucuronides and their parent drugs eluted within 9 min (Figure 5).

Positive electrospray ionization (ESI+) was used for most analytes (Supplementary Tables S1 and S2). The following source conditions were used: capillary voltage 3.0 kV, source temperature 150 °C, desolvation temperature 600 °C, cone gas flow 150 L/h and desolvation gas flow 1000 L/h. Argon was used as the collision gas at a flow of 0.14 mL/minute corresponding to a pressure of 4.2 × 10^−3^ in the collision cell. The mass spectrometer was operated in the multiple-reaction monitoring (MRM) mode. MRM transitions, collision energies and retention times (RT) for each analyte and IS are shown in Supplementary Table S1. An additional 141 analytes relevant for DFSA were included to investigate stability under the different temperature and incubation setups. A complete list of these is provided in Supplementary Table S2. Cone voltage was 20 V for all analytes and internal standards. MassLynx 4.2 SCN 986 software with TargetLynx (Waters Corporation, Milford, MA, USA) was used for data acquisition and data processing.

### 3.8. Estimation of Hydrolysis Efficiency

The hydrolysis efficiency was estimated based on how fast ≥90% glucuronide cleavage was obtained under conditions based on the manufacturer’s standard procedure. The time frame to obtain ≥90% glucuronide cleavage was divided into five intervals: very fast (≤5 min), fast (≤10 min), medium (≤60 min), slow (≤18 h) and very slow (≥24 h). An efficient hydrolysis was defined as ≥90% glucuronide cleavage within the shortest amount of time.

The stability of drugs and metabolites was estimated from the difference between peak areas before and after incubation.

### 3.9. Ethical Considerations

The study was a method development study with applications in anonymized human drug-free urine and no identifiable data were included in the study. The study was registered and complied with the European General Data Protection Regulation (Regulation number 2016/697, Journal number: 514-0911/23-3000).

## 4. Conclusions

The hydrolysis efficiency of three β-glucuronidases was evaluated on 11 conjugated metabolites from drugs relevant to DFSA. The recombinant β-glucuronidase B-One™ demonstrated the best overall hydrolysis efficiency with a very short incubation time (5–10 min) at room temperature. The recombinant β-glucuronidase BGTurbo™ showed similar efficiency but with ranging temperatures (20–55 °C) and a longer incubation time (5–60 min), while the β-glucuronidase/arylsulfatase (*H. pomatia*) required heating at the longest incubation period (18–24 h) to achieve a similar efficiency. Additionally, the stability of 99% of 141 drugs and metabolites relevant to DFSA was not affected by incubation for 24 h at 20 °C, 40 °C and 55 °C, which indicates no considerable difference between using enzymes that require heating or not. The choice of enzyme involves considerations of the target drugs as well as the specific requirements, workflow, and economy of the laboratory; but, ultimately, the use of recombinant enzymes shows the rapid and efficient hydrolysis of a broad panel of glucuronides relevant to DFSA. Additionally, the use of recombinant enzymes enables a simple and optimized workflow for the pretreatment of urine samples in systematic toxicological analyses of urine samples in DFSA cases.

## Data Availability

The data presented in this study are available in the Supplementary Materials.

## Supplementary Materials

The following supporting information can be downloaded at: https://www.mdpi.com/article/10.3390/ph17010013/s1, Figure S1: Cleavage (%) of all 11 glucuronides when enzymatically hydrolyzed with either MIX, B-One or BGT at 20 °C, 40 °C and 55 °C for 24 h. The *x*-axis is in categorical order based on the five incubation periods; Figure S2: Analyte recovery (%) of the 10 parent drugs when their corresponding glucuronide is enzymatically hydrolyzed with either MIX, B-One or BGT at 20 °C, 40 °C and 55 °C for 24 h. The *x*-axis is in categorical order based on the five incubation periods; Table S1: Retention time (RT), MRM transitions, collision energies, internal standards (IS) and ionization mode for the 11 glucuronides and their parent drug; Table S2: Retention time (RT), MRM transitions, collision energies, internal standards (IS) and ionization mode for the 141 drugs and metabolites included in the stability experiment; Table S3: Analyte recovery (%) for the 11 glucuronides and their parent drug after incubation with each enzyme at 20 °C for the five incubation periods. Table S4: Analyte recovery (%) for the 11 glucuronides and their parent drug after incubation with each enzyme at 40 °C for the five incubation periods; Table S5: Analyte recovery (%) for the 11 glucuronides and their parent drug after incubation with each enzyme at 55 °C for the five incubation periods.
