# Supplementary material for: Exploring Enzymatic Hydrolysis of Urine Samples for Investigation of Drugs Associated with Drug-Facilitated Sexual Assault
Source: Pharmaceuticals (Basel). 2023 Dec 21;17(1):13. doi: 10.3390/ph17010013 (PMC10818341; doi:10.3390/ph17010013)
Supplement: Supplementary file 1 [file pharmaceuticals-17-00013-s001.zip › pharmaceuticals-2762993-supplementary.pdf]

Supplementary Table S1 Retention time (RT), MRM transitions, collision energies, internal standards (IS) and ionization mode for the 11 glucuronides and their parent drug.

Analyte	RT (min)	MRM 1 (m/z)	CE 1 (eV)	MRM 2 (m/z)	CE 2 (eV)	Internal standard	MRM 1 (m/z)	Ionization mode
Amitriptyline	7.67	278 > 233	16	278 > 91	24	Amitriptyline-d6	284 > 91	ESI+
Amitriptyline glucuronide	7.39	454 > 233	18	454 > 105	32	Amitriptyline-d6	284 > 91	ESI+
Buprenorphine	7.26	468 > 84	40	468 > 55	40	Amitriptyline-d6	284 > 91	ESI+
Buprenorphin glucuconide	6.39	644 > 468	42	644 > 414	50	Amitriptyline-d6	284 > 91	ESI+
Codeine	3.52	300 > 165	48	300 > 215	24	Codeine-d6	306 > 218	ESI+
Codeine-6-glucuronide	3.32	476 > 300	28	476 > 282	22	Codeine-d6	306 > 218	ESI+
Diphenhydramine	6.98	256 > 167	12	256 > 152	36	Amitriptyline-d6	284 > 91	ESI+
Diphenhydramine glucuronide	6.82	432 > 256	20	432 > 167	24	Amitriptyline-d6	284 > 91	ESI+
Doxylamine	4.67	271 > 182	20	271 > 167	38	Morphine-d6	292 > 201	ESI+
Doxylamine glucuronide	4.15	447 > 226	16	447 > 182	30	Morphine-d6	292 > 201	ESI+
Lorazepam	7.94	323 > 231	29	323 > 229	29	Amitriptyline-d6	284 > 91	ESI+
Lorazepam glucuronide	7.31	496 > 320	12	496 > 274	36	Amitriptyline-d6	284 > 91	ESI+
Morphine	2.31	286 > 201	24	286 > 165	38	Morphine-d6	292 > 201	ESI+
Morphine-3-glucuronide	1.82	462 > 285	26	462 > 152	74	Morphine-d6	292 > 201	ESI+
Morphine-6-glucuronid	2.31	462 > 286	30	462 > 201	40	Morphine-d6	292 > 201	ESI+
Oxymorphone	3.49	302 > 227	28	302 > 198	42	Morphine-d6	292 > 201	ESI+
Oxymorphone-3-glucuronide	1.81	478 > 284	24	478 > 227	42	Morphine-d6	292 > 201	ESI+
Scopolamin	3.87	304 > 138	20	304 > 156	14	Morphine-d6	292 > 201	ESI+
Scopolamine glucuronide	3.71	478 > 175	18	478 > 113	22	Morphine-d6	292 > 201	ESI+
Temazepam	8.23	301 > 177	36	301 > 255	22	Temazepam-d5	306 > 260	ESI+
Temazepam glucuronide	7.51	477 > 301	15	477 > 283	25	Temazepam-d5	306 > 260	ESI+

Supplementary Table S2 Retention time (RT), MRM transitions, collision energies, internal standards (IS) and ionization mode for the 141 drugs and metabolites included in the stability experiment.

Analyte	RT (min)	MRM 1 (m/z)	CE 1 (eV)	MRM 2 (m/z)	CE 2 (eV)	Ionization mode
10,11-Dihydro-10-hydroxycarbazepine	6.55	255 > 237	8	255 > 194	18	ESI+
6-MAM	4.01	328 > 165	26	328 > 211	34	ESI+
7-Aminoclonazepam	5.06	286 > 222	24	286 > 121	30	ESI+
7-Aminoflunitrazepam	5.86	284 > 135	26	284 > 227	25	ESI+
7-Aminonitrazepam	3.53	252 > 121	20	252 > 94	36	ESI+
9-Hydroxyrisperidone	6.23	427 > 207	27	427 > 110	49	ESI+
Acepromazine	2.04	129 > 75	26	129 > 93	16	ESI+
Alpha-hydroxyalprazolam	7.83	325 > 243	26	325 > 279	34	ESI+
Alpha-hydroxymidazolam	7.12	342 > 16	42			ESI+
Alprazolam	8.10	309 > 281	24	309 > 205	38	ESI+
Amphetamine	3.82	136 > 119	6	136 > 91	14	ESI+
Amisulpride	4.59	370 > 242	26	370 > 196	38	ESI+
Amitriptyline	7.67	278 > 233	16	278 > 91	24	ESI+
Amitriptyline glucuronide	7.39	454 > 233	18	454 > 105	32	ESI+
Amlodipine	7.67	409 > 238	11	409 > 294	13	ESI+
Aripiprazole	7.60	448 > 285	26	448 > 176	31	ESI+
Atropine	4.73	290 > 124	25	290 > 103	50	ESI+
Baclofen	4.05	214 > 116	29	214 > 151	18	ESI+
Benzoylecgonine	5.01	290 > 105	30	290 > 168	18	ESI+
Bromazepam	7.27	318 > 182	30	318 > 209	25	ESI+
Buprenorphine	7.26	468 > 84	40	468 > 55	40	ESI+
Buprenorphine glucuronide	6.39	644 > 468	42	644 > 414	50	ESI+
Carbamazepine	7.59	237 > 194	18	237 > 179	34	ESI+
Carbamazepine-10,11-epoxide	6.86	253 > 180	24	253 > 210	14	ESI+
Carisoprodol	7.91	261 > 55	28	261 > 176	10	ESI+
Cathine	3.03	152 > 134	8	152 > 117	16	ESI+
Cathinone	3.10	150 > 117	18	150 > 105	16	ESI+
Cetirizine	7.80	389 > 201	19	389 > 166	63	ESI+
Chlordiazepoxide	5.03	300 > 227	24	300 > 283	23	ESI+
Chlorpheniramine	6.41	275 > 230	16	275 > 167	36	ESI+
Chlorprothixene	7.98	316 > 231	28	316 > 271	18	ESI+
Cinnarizine	8.24	369 > 167	19	369 > 152	53	ESI+
Citalopram	7.02	325 > 109	26	325 > 262	18	ESI+

Analyte	RT (min)	MRM 1 (m/z)	CE 1 (eV)	MRM 2 (m/z)	CE 2 (eV)	Ionization mode
Clemastine	8.21	344 > 215	18	344 > 130	10	ESI+
Clobazam	8.17	301 > 224	32	301 > 259	20	ESI+
Clomipramine	8.01	315 > 86	18	315 > 58	32	ESI+
Clonazepam	7.76	316 > 270	24	316 > 214	35	ESI+
Clozapine	6.83	327 > 270	22	327 > 192	40	ESI+
Cocaine	5.80	304 > 82	26	304 > 182	18	ESI+
Codeine	3.52	300 > 165	48	300 > 215	24	ESI+
Codeine-6-glucuronide	3.32	476 > 300	28	476 > 282	22	ESI+
Cyclizine	7.07	267 > 167	15	267 > 152	39	ESI+
Demoxepam	7.45	287 > 179	20	287 > 105	20	ESI+
Dextromethorphane	6.95	272 > 171	38	272 > 215	22	ESI+
Dextropropoxyphene	7.62	340 > 58	14	340 > 266	12	ESI+
Diazepam	8.61	285 > 154	26	285 > 193	30	ESI+
Dihydrocodeine	3.49	302 > 199	30	302 > 227	25	ESI+
Diphenhydramine	6.98	256 > 167	12	256 > 152	36	ESI+
Diphenhydramine glucuronide	6.82	432 > 256	20	432 > 167	24	ESI+
Doxylamine	4.67	271 > 182	20	271 > 167	38	ESI+
Doxylamine glucuronide	4.15	447 > 226	16	447 > 182	30	ESI+
Fenazepam	8.40	350 > 206	36	348 > 183	30	ESI+
Fentanyl	6.90	337 > 188	23	337 > 105	41	ESI+
Fexofenadine	7.63	502 > 466	27	502 > 171	37	ESI+
Flunitrazepam	7.95	314 > 239	34	314 > 268	25	ESI+
Fluoxetine	7.74	310 > 44	14	310 > 148	9	ESI+
Flupentixol	8.36	435 > 265	14	435 > 100	9	ESI+
Gabapentin	3.65	172 > 154	16	172 > 137	10	ESI+
GHB	1.35	103 > 57	20	103 > 85	8	ESI-
Haloperidol	7.21	376 > 165	24	376 > 123	36	ESI+
Hydrocodone	3.52	300 > 199	28	300 > 171	38	ESI+
Hydromorphone	2.75	286 > 185	28	286 > 157	39	ESI+
Hydroxizine	7.61	375 > 201	20	375 > 166	60	ESI+
Imipramine	7.56	281 > 86	10	281 > 208	25	ESI+
Ketamine	5.06	238 > 125	24	238 > 179	15	ESI+
Ketobemidone	4.49	248 > 190	28	248 > 70	32	ESI+
Lamotrigine	5.26	256 > 210	25	256 > 171	35	ESI+
Levetiracetam	3.51	171 > 126	12	171 > 69	26	ESI+
Levomepromazine	7.70	329 > 100	18	329 > 242	22	ESI+

Analyte	RT (min)	MRM 1 (m/z)	CE 1 (eV)	MRM 2 (m/z)	CE 2 (eV)	Ionization mode
Lorazepam	7.94	323 > 231	29	323 > 229	29	ESI+
Lorazepam glucuronide	7.31	496 > 320	12	496 > 274	36	ESI+
Lormetazepam	8.36	335 > 289	21	335 > 177	42	ESI+
MDA	4.01	180 > 163	8	180 > 105	20	ESI+
MDMA	4.22	194 > 105	20	194 > 163	12	ESI+
Meclozine	8.51	391 > 201	21	391 > 165	43	ESI+
Melatonin	6.26	233 > 174	15	233 > 159	27	ESI+
Meprobamate	6.48	219 > 158	8	219 > 97	24	ESI+
Metamphetamine	4.09	150 > 119	10	150 > 91	18	ESI+
Methadone	7.74	310 > 105	26	310 > 265	14	ESI+
Methylphenidate	5.62	234 > 84	19	234 > 56	36	ESI+
Metoclopramide	5.03	300 > 227	16	300 > 184	28	ESI+
Metoprolol	5.63	268 > 116	14	268 > 74	16	ESI+
Mianserin	6.98	265 > 58	20	265 > 208	20	ESI+
Midazolam	7.01	326 > 291	26	326 > 223	36	ESI+
Mirtazapine	5.47	266 > 195	26	266 > 72	18	ESI+
Morphine	2.31	286 > 201	24	286 > 165	38	ESI+
Morphine-3-glucuronide	1.82	462 > 285	26	462 > 152	74	ESI+
Morphine-6-glucuronide	1.82	462 > 286	30	462 > 201	40	ESI+
N-Demethylclozapine	6.60	313 > 192	41	313 > 270	25	ESI+
N-Demethyldiazepam	8.20	271 > 140	28	271 > 165	28	ESI+
Nitrazepam	7.67	282 > 236	22	282 > 180	35	ESI+
Norbuprenorphine	6.80	414 > 83	36	414 > 101	44	ESI+
Norclobazam	7.77	287 > 245	22	287 > 210	32	ESI+
Norfentanyl	5.11	233 > 84	16	233 > 150	16	ESI+
Norfluoxetine	4.73	296 > 134	4	296 > 296	2	ESI+
Norketamine	4.94	224 > 125	24	224 > 207	13	ESI+
Nortriptyline	7.67	264 > 233	12	264 > 91	14	ESI+
O-Desmethyltramadol	4.21	250 > 58	12	250 > 232	13	ESI+
O-Desmethylvenlafaxine	5.10	264 > 107	31	264 > 133	24	ESI+
Olanzapine	3.87	313 > 256	22	313 > 84	22	ESI+
Ondansetron	5.99	294 > 170	26	294 > 184	26	ESI+
Oxazepam	7.87	287 > 241	32	287 > 104	22	ESI+
Oxazepam glucuronide	6.98	463 > 287	20	463 > 269	25	ESI+
Oxcarbazepine	7.06	253 > 208	20	253 > 180	28	ESI+
Oxycodone	3.83	316 > 241	30	316 > 298	18	ESI+
Oxymorphone	3.49	302 > 227	28	302 > 198	42	ESI+
Oxymorphone-3-glucuronide	1.81	478 > 284	24	478 > 227	42	ESI+

Analyte	RT (min)	MRM 1 (m/z)	CE 1 (eV)	MRM 2 (m/z)	CE 2 (eV)	Ionization mode
Paroxetine	7.38	330 > 192	24	330 > 70	28	ESI+
Pentobarbital	7.54	225 > 182	14	225 > 42	20	ESI-
Perphenazine	8.02	404 > 171	22	404 > 143	28	ESI+
Pethidine	6.03	248 > 220	20	248 > 174	16	ESI+
Phenobarbital	6.53	231 > 188	10	231 > 42	14	ESI-
Phenytoin	7.41	253 > 182	16	253 > 104	30	ESI+
Pregabalin	3.59	160 > 55	18	160 > 97	12	ESI+
Primidone	5.45	219 > 91	28	219 > 162	12	ESI+
Promethazine	7.34	285 > 198	24	285 > 240	15	ESI+
Propranolol	6.80	260 > 116	17	260 > 56	29	ESI+
Quetiapine	7.02	384 > 221	38	384 > 253	22	ESI+
Reboxetine	7.25	314 > 176	11	314 > 91	31	ESI+
Risperidone	6.47	411 > 82	26	411 > 191	58	ESI+
Scopolamine	3.87	304 > 138	20	304 > 156	14	ESI+
Scopolamine glucuronide	3.71	478 > 175	18	478 > 113	22	ESI-
Sertraline	7.91	306 > 158	12	308 > 277	24	ESI+
Sulpirid	3.15	342 > 214	32	342 > 112	24	ESI+
Suvorexant	9.46	450 > 186	22	450 > 104	62	ESI+
Tapentadol	5.74	222 > 121	20	222 > 107	24	ESI+
Temazepam	8.23	301 > 177	36	301 > 255	22	ESI+
Temazepam glucuronide	7.51	477 > 301	15	477 > 283	25	ESI+
Thiopental	8.09	241 > 57	16	241 > 100	14	ESI-
Topiramate	6.99	340 > 264	6	340 > 184	12	ESI+
Tramadol	5.60	264 > 58	12	264 > 246	10	ESI+
Trazodone	6.51	372 > 148	35	372 > 176	25	ESI+
Triazolam	8.12	343 > 308	24	343 > 239	40	ESI+
Venlafaxine	6.59	278 > 121	28	278 > 91	40	ESI+
Zaleplon	7.50	306 > 236	26	306 > 264	20	ESI+
Ziprasidone	6.92	413 > 159	40	413 > 194	26	ESI+
Zolpidem	6.29	308 > 235	36	308 > 92	52	ESI+
Zonisamide	5.16	213 > 132	14	213 > 76	28	ESI+
Zopiclone	5.36	389 > 245	16	389 > 217	34	ESI+
Zuclopenthixol	8.14	401 > 231	41	401 > 128	25	ESI+

Supplementary Table S3 Analyte recovery (%) for the 11 glucuronides and their parent drug after incubation with each enzyme at 20°C for the five incubation periods.

	**20°C**														
	**MIX**					**B-One**					**BGT**				
**ANALYTE**	**5 min**	**10 min**	**60 min**	**18 h**	**24 h**	**5 min**	**10 min**	**60 min**	**18 h**	**24 h**	**5 min**	**10 min**	**60 min**	**18 h**	**24 h**
AMITRIPTYLINE	1	1	1	6	8	98	99	100	100	98	28	72	83	80	84
AMITRIPTYLINE GLUCURONIDE	55	52	51	47	44	1	1	1	0	0	59	8	0	0	0
BUPRENORPHINE	5	8	27	43	43	60	61	57	65	56	42	72	72	79	75
BUPRENORPHINE GLUCUCONIDE	38	38	16	0	0	0	0	0	0	0	29	2	0	0	0
CODEINE	1	1	1	10	11	93	103	119	126	117	4	23	64	99	88
CODEINE-6-GLUCURONIDE	103	85	83	81	74	14	9	0	0	0	87	77	30	0	0
DIPHENHYDRAMINE	1	2	2	11	13	103	96	97	94	101	44	100	100	97	100
DIPHENHYDRAMINE GLUCURONIDE	49	47	46	38	36	1	1	1	0	0	61	9	0	0	0
DOXYLAMINE	2	2	2	6	7	36	35	39	38	39	7	29	38	40	40
DOXYLAMINE GLUCURONIDE	83	75	80	70	73	1	1	0	0	0	72	21	0	0	0
LORAZEPAM	7	10	31	46	49	59	63	61	55	60	41	79	78	79	78
LORAZEPAM GLUCURONIDE	50	44	19	1	2	2	2	2	2	2	47	5	2	2	2
MORPHINE	3	3	11	57	63	70	73	89	92	92	6	32	59	83	82
MORPHINE-3-GLUCURONIDE	102	87	73	4	2	0	0	0	0	0	78	40	1	0	0
MORPHINE-6-GLUCURONIDE	71	61	65	38	34	34	29	4	0	0	83	86	62	1	0
OXYMORPHONE	1	1	2	13	14	14	15	16	16	16	1	7	20	23	23
OXYMORPHONE-3-GLUCURONIDE	81	70	68	10	6	4	2	0	0	0	89	70	7	0	0
SCOPOLAMINE	4	5	19	54	56	69	70	70	71	71	14	68	91	94	91
SCOPOLAMINE GLUCURONIDE	96	87	69	0	0	1	1	1	1	0	79	27	0	0	0
TEMAZEPAM	14	17	54	99	99	110	112	110	111	114	26	89	107	106	107
TEMAZEPAM GLUCURONIDE	131	116	81	0	0	0	0	0	0	0	69	35	0	0	0

Supplementary Table S4 Analyte recovery (%) for the 11 glucuronides and their parent drug after incubation with each enzyme at 40°C for the five incubation periods.

	**40°C**														
	**MIX**					**B-One**					**BGT**				
**ANALYTE**	**5 min**	**10 min**	**60 min**	**18 h**	**24 h**	**5 min**	**10 min**	**60 min**	**18 h**	**24 h**	**5 min**	**10 min**	**60 min**	**18 h**	**24 h**
AMITRIPTYLINE	1	1	1	2	32	38	99	104	98	96	68	72	75	76	77
AMITRIPTYLINE GLUCURONIDE	48	48	45	23	21	1	1	1	0	0	12	3	0	0	0
BUPRENORPHINE	11	24	43	39	40	56	69	62	62	54	67	66	65	70	66
BUPRENORPHINE GLUCUCONIDE	34	24	1	0	0	0	0	0	0	0	2	0	0	0	0
CODEINE	1	1	3	47	51	91	104	109	109	117	18	27	79	87	86
CODEINE-6-GLUCURONIDE	89	74	66	33	24	12	5	0	0	0	72	66	3	0	0
DIPHENHYDRAMINE	1	2	4	37	41	94	105	102	85	90	92	87	99	87	89
DIPHENHYDRAMINE GLUCURONIDE	45	46	41	14	10	1	1	1	0	0	14	4	0	0	0
DOXYLAMINE	2	2	2	24	27	36	36	39	39	41	23	29	35	38	38
DOXYLAMINE GLUCURONIDE	79	79	75	46	39	1	1	0	0	0	31	13	1	0	0
LORAZEPAM	11	22	41	40	41	58	59	50	49	46	79	72	79	65	64
LORAZEPAM GLUCURONIDE	37	24	1	1	1	2	2	1	2	2	5	1	2	1	2
MORPHINE	4	9	31	81	84	70	77	98	91	91	26	34	71	80	78
MORPHINE-3-GLUCURONIDE	77	62	31	0	0	0	0	0	0	0	46	24	0	0	0
MORPHINE-6-GLUCURONIDE	57	55	49	2	1	34	23	0	0	0	84	75	21	0	0
OXYMORPHONE	1	2	7	15	15	15	16	14	17	17	5	8	21	22	21
OXYMORPHONE-3-GLUCURONIDE	66	61	33	0	0	4	1	0	0	0	73	55	0	0	0
SCOPOLAMINE	6	13	37	55	55	66	68	56	73	73	52	68	86	89	85
SCOPOLAMINE GLUCURONIDE	83	63	27	0	0	1	1	1	0	0	40	18	0	0	0
TEMAZEPAM	21	39	96	100	94	111	113	121	112	113	76	91	99	104	101
TEMAZEPAM GLUCURONIDE	115	86	0	0	0	0	0	0	0	0	43	30	0	0	0

Supplementary Table S5 Analyte recovery (%) for the 11 glucuronides and their parent drug after incubation with each enzyme at 55°C for the five incubation periods.

	55°C														
	**MIX**					**B-One**					**BGT**				
**ANALYTE**	**5 min**	**10 min**	**60 min**	**18 h**	**24 h**	**5 min**	**10 min**	**60 min**	**18 h**	**24 h**	**5 min**	**10 min**	**60 min**	**18 h**	**24 h**
AMITRIPTYLINE	1	1	1	5	59	67	93	92	94	91	80	80	79	78	77
AMITRIPTYLINE GLUCURONIDE	54	51	48	11	6	1	1	1	0	0	1	1	0	0	0
BUPRENORPHINE	7	14	41	40	41	57	58	58	54	53	75	88	80	77	72
BUPRENORPHINE GLUCUCONIDE	37	32	0	0	0	0	0	0	0	0	0	0	0	0	0
CODEINE	1	1	6	60	75	84	106	110	109	115	20	36	83	91	91
CODEINE-6-GLUCURONIDE	76	97	81	17	12	16	6	0	0	0	72	61	1	0	0
DIPHENHYDRAMINE	1	2	7	47	49	91	102	96	77	86	113	128	108	97	95
DIPHENHYDRAMINE GLUCURONIDE	50	46	43	7	4	1	1	1	0	0	1	1	0	0	0
DOXYLAMINE	2	2	4	36	42	34	35	38	41	41	31	35	38	39	39
DOXYLAMINE GLUCURONIDE	82	88	87	32	23	1	1	0	0	0	9	1	0	0	0
LORAZEPAM	8	17	49	36	36	58	62	53	33	31	84	93	79	51	45
LORAZEPAM GLUCURONIDE	48	32	2	2	1	2	2	2	1	1	2	2	2	1	1
MORPHINE	3	5	50	90	92	63	74	87	88	87	33	47	80	83	83
MORPHINE-3-GLUCURONIDE	82	80	14	0	0	0	0	0	0	0	33	7	0	0	0
MORPHINE-6-GLUCURONID	63	67	55	0	0	34	22	0	0	0	87	81	10	1	1
OXYMORPHONE	1	1	12	17	17	14	15	16	16	16	7	13	23	24	23
OXYMORPHONE-3-GLUCURONIDE	71	75	20	0	0	5	0	0	0	0	68	42	0	0	0
SCOPOLAMINE	4	7	54	60	62	61	64	67	66	64	77	85	90	89	82
SCOPOLAMINE GLUCURONIDE	90	88	12	0	0	1	1	0	0	0	12	1	0	1	0
TEMAZEPAM	15	30	103	110	110	110	108	109	108	107	103	104	106	105	105
TEMAZEPAM GLUCURONIDE	122	100	0	0	0	0	0	0	0	0	0	0	0	0	0

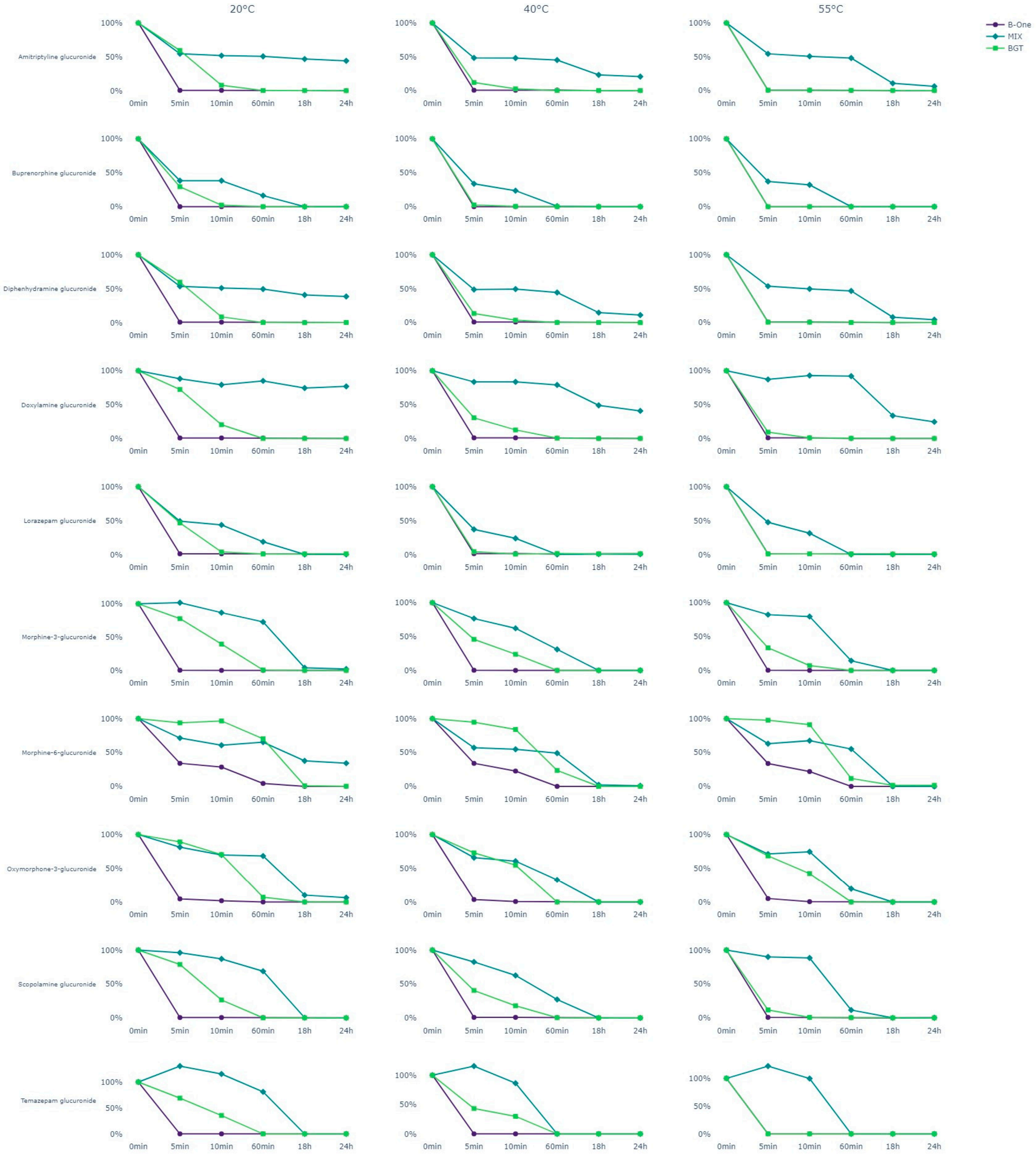

Supplementary Figure S1 Cleavage (%) of 10 glucuronides when enzymatically hydrolyzed with either MIX, B-One or BGT at 20°C, 40°C and 55°C for 24 hours. The x-axis is in categorical order based on the five incubation periods.

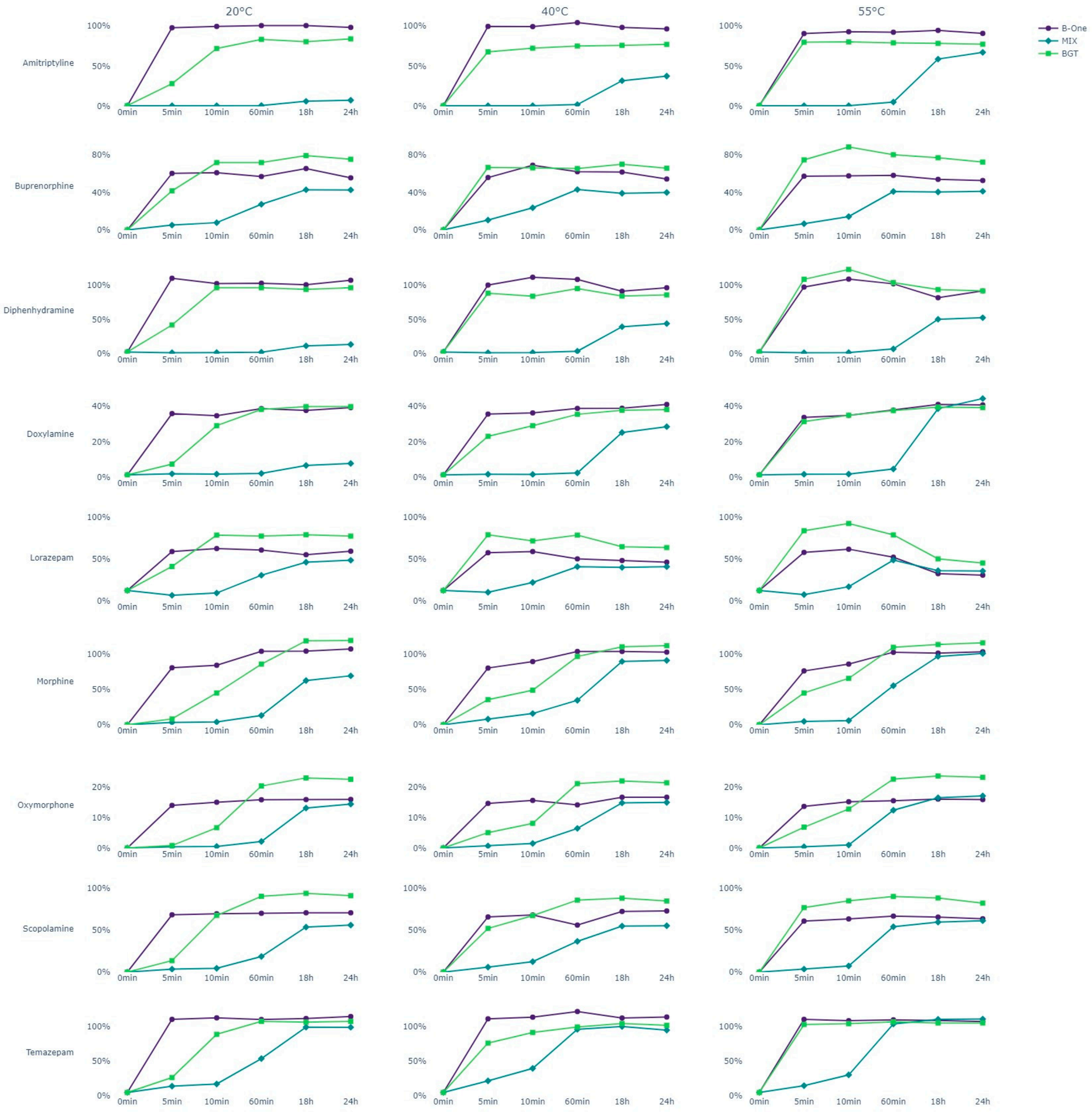

Supplementary Figure S2 Analyte recovery (%) of 9 parent drugs when their corresponding glucuronide is enzymatically hydrolyzed with either MIX, B-One or BGT at 20°C, 40°C and 55°C for 24 hours. The x-axis is in categorical order based on the five incubation periods.
